# Tensions Between Ethics and the Law: Examination of a Legal Case by Two Midwives Invoking a Conscientious Objection to Abortion in Scotland

**DOI:** 10.1007/s10730-019-09378-4

**Published:** 2019-07-04

**Authors:** Valerie Fleming, Lucy Frith, Beate Ramsayer

**Affiliations:** 1 School of Nursing and Allied Health, Liverpool John Moores, University, 15-21 Webster St, Liverpool, L3 2ET UK; 2 University of Liverpool, Liverpool, UK

**Keywords:** Abortion, Midwife, Women’s health

## Abstract

This paper examines a legal case arising from a workplace grievance that progressed to being heard at the UK’s Supreme Court. The case of Doogan and Wood versus Greater Glasgow and Clyde Health Board concerned two senior midwives in Scotland, both practicing Roman Catholics, who exercised their perceived rights in accordance with section 4(1) of the Abortion Act not to participate in the treatment of women undergoing abortions. The key question raised by this case was: “Is Greater Glasgow and Clyde Health Board entitled to require the midwives to delegate, supervise and support staff in the treatment of patients undergoing termination of pregnancy?” The ethical issues concerning conscientious objection to abortion have been much debated although the academic literature is mainly concerned with the position of medical practitioners rather than what the World Health Organization terms “mid-level professionals” such as midwives. This paper examines the arguments put forward by the midwives to justify their refusal to carry out tasks they felt contravened their legal right to make a conscientious objection. We then consider professional codes, UK legislation and church legislation. While the former are given strong weighting the latter was been ignored in this case, although cases in other European countries have been prevented from escalating to such a high level by the intervention of prominent church figures. The paper concludes by stating that the question put to the courts remains as yet unanswered but offers some recommendations for future policy making and research.

## Background

In 69% of the world’s developed countries, induced termination of pregnancy (abortion) is available on demand with an estimated 56 million abortions taking place in 2014 (Gutmacher Institute 2018). Due to a shortage of medical practitioners, it is to midwives that many countries are turning to provide safe abortion services. This is strongly supported by the World Health Organization (WHO) which recommends “mid-level professionals” such as midwives or nurses as the key providers in the provision of abortion services (World Health Organization 2012). Likewise, the International Confederation of Midwives’ (ICM) (2014) essential competencies for practice, include provision of safe abortion care.

While much of the work of the United Nations (UN), WHO and ICM focuses on developing countries, in England, Wales and Scotland legalized abortion, under the supervision of medical practitioners, is currently permitted up to the completion of week 24 of pregnancy when certain criteria are fulfilled (Abortion Act 1967). Care for women having abortions taking place after approximately the 16th week of pregnancy is mainly provided by midwives in the safe environment of a hospital labor ward. Section 4(1) of the 1967 Abortion Act provides that no one is under any duty to participate, contrary to his or her conscience, in any treatment authorized by the Act, although the exemption does not apply where treatment “is necessary to save the life or to prevent grave permanent injury to the physical or mental health of a pregnant woman” (Abortion Act, Section 4(2)). In practice, conscientious objection to abortion often becomes the center of acrimonious debate, with practitioners as well as academics polarized as to the rights and wrongs both of the procedure and of health care professionals’ objections to participating in the procedure.

This paper is not about the rights or wrongs of abortion, it is about conscientious objection to abortion. We will examine the legal case of two Scottish midwives, who exercised their perceived rights in accordance with Section 4(1) of the Abortion Act not to participate in the treatment of women undergoing abortions. We will use this case to assess how the legal authorities in Scotland and the UK decided what constitutes participation in an abortion and to what elements of procedures and care should one be able to conscientiously object. What delineates participating in abortion and the moral significance of different forms of proximity to the abortion act are contested issues, and part of the dispute was exactly what the midwives had a right to conscientiously object *to*, with the two sides disagreeing over what constituted such participation and hence what activities were covered by the Act. We also consider the weight given to religious views of the conscientious objectors—should objection based on such views be given more or less weight? The case of Doogan and Wood versus the Greater Glasgow and Clyde Health Board (GGCHB) is traced from its origins in one clinical area in Glasgow, Scotland, in 2005 through to the judgment of the UK Supreme Court on 17 December 2014. The paper focuses on the question raised in the hearings, “Is GGCHB entitled to require the midwives to delegate, supervise and support staff in the treatment of patients undergoing termination of pregnancy?”

As the two midwives concerned were practicing Roman Catholics, the laws of the Catholic Church, which prohibit abortion for any reason, are also considered in this paper. The Church has held a constant position on the sanctity of human life with Pope Pius XII (1951) over half a century ago, notably addressing Italian midwives specifically about their role in relation to abortion. At the same time as politicians in the UK were lobbying for or against the Abortion Bill, Pope Paul VI (1968) promulgated the Encyclical *Humanae Vitae* (HV), which makes clear the Church’s absolute veto on abortion.

*The Code of Canon Law* (CIC) (1983), building on the theological developments taking place from the Second Vatican Council onwards, lays out in two paragraphs what it considers to be some of the more serious delicts in relation to human life. In this, Canon 1398 states that “anyone who procures a completed abortion incurs a *latae sentiae* [automatic] excommunication” (*Code of Cannon Law* 1983). For those who adhere to the Roman Catholic faith, this canon is of extreme importance and was highly influential for the two midwives at the center of this case, who would have been excommunicated if they had participated in abortion-related care. This paper, while primarily examining the legal case, considers the possible positioning of the conscience clause in relation to published literature in an effort to seek the clarity that the legal judgement failed to provide.

### Relevant Literature

There is a vast body of published literature on the subject of conscientious objection in relation to the provision of health services.^1^ Abortion accounts for an estimated 80% of these articles but this literature does not appear to have been taken into account by the judges in this case as none of the academic debates were discussed or cited in any of the judgements. This article provides an overview of debates over: conscience and conscientious objection; conscientious objection to abortion; and midwifery and conscientious objection, before the case is presented.

### Conscience and Conscientious Objection

Sulmasy (2008) makes the point that despite many debates on the issue of conscience, insufficient attention has been paid to understanding what conscience actually is and what its importance might be. This is not strictly the case; the philosophical concept of conscience has been debated for several millennia with Aristotle, in outlining his philosophy of a virtuous life, stating that a mature conscience allows persons who know what they are doing to act virtuously (Caston 2004). The Aristotelian conceptualization of conscience lacks the sense of humans as beings essentially being immersed in a struggle between good and evil. This later emerges vividly in the Judaeo-Christian worldview in which conscience is portrayed as the antithesis of a sin.

In the letters of Paul and several other places in the New Testament, as well as in some non-Christian writers of roughly the same period, the first occurrences of an explicit term depicting the concept of conscience are articulated. In a frequently cited passage Paul remarks that the Gentiles, though not possessing the Law, “can point to the substance of the Law engraved on their hearts – they can call a witness, that is, their own conscience—they have accusation and defense, that is, their own inner mental dialogue” (Romans 2:15).

It was, however, St. Thomas Aquinas [ca. (1270)] who developed the notion of conscience more fully concluding that conscience is not a power but an act as by its very nature it implies the relation of knowledge to individual cases. Such was to be the basis for a considerable body of philosophers’ works over successive centuries. In the 20th century and beyond, the “act” in Aquinas’ conceptualization, is commonly to be found in the health care setting. Wicclair (2000) made a link between conscience and integrity, including toleration of diversity, respect for autonomy, and respect for moral integrity as the grounds for protecting the notion of conscience. Wicclair concludes that *carte blanche* rights of conscientious objection should not be given but rather respected for the moral integrity of the physician is the best way forward. In this way, he argues “appeals to conscience can be understood as efforts to preserve or maintain moral integrity” (Wicclair 2000, p. 213). A decade later Wicclair’s position was supported by Antommaria (2010), who reasoned that conscience needs to be understood as synonymous with the maintenance of personal integrity. Any claims of conscientious objection thus derive from the importance of the value of integrity and subsequent moral agency underpinning them. Sulmasy’s view when applied to the majority of writers who analyze conscience in the healthcare context is relevant, as generally they do not clarify the connection between conscience, integrity and moral agency. Some, however, do: Weinstock (2014) for example, in discussing the influence of religion on conscience, comments that if a health professional’s right to conscientiously object is respected, “respect [is afforded to] the moral agency of those who hold reasonable dissenting views” (p. 12). In the same vein, he points out that a state which neither protected conscience nor permitted individuals to act according to the conclusions of their moral reasoning would fail to display appropriate respect for them as moral agents. In a later paper, Sulmasy (2017, p. 22) emphasizes the point that because a service, e.g., abortion, is legal, it does not compel a physician (or other health professionals) to participate in it. Instead, he argues that all professionals should be afforded a discretionary space and that general conditions of tolerance should prevail. Tolerance springs from humility, which he explained as “honest acknowledgement that one´s moral judgements are fallible” (p. 22).

Such debates among philosophers and ethicists may initially appear to be far removed from the realities of the present case. However, some writers bring this closer reflecting on the “rampant spread” of conscientious objection in health care settings (Zampas et al. 2013), while others challenge the rights of health care professionals to allow their private values to interfere with their work (Baker 2009). Neal (2015) counters these views, adopting the generally accepted view of conscience in bioethics posed by Wicclair (2000) and seeing the faculty of conscience as a fundamental feature of all areas of human endeavor, including professional practice. Neal thus asks how conscience can be protected. Contrary to the views of Zampas and other authors, she suggests that the apparent expansion of conscientious objection claims is based on poorly defined or even contradictory professional guidelines. Drawing on the legal literature, Neal proposes a need for research establishing working definitions and concludes that there are four criteria for a justifiable position of conscientious objection:

1. The position must be sincere (the “sincerity criterion”),
2. The health professional seeking the conscience-based exemption (CBE) must be able to articulate the basis of her position (the “articulation criterion”),
3. The position must not be intolerant and must not disrespect the conscientious position of others (the “tolerance/respect criterion”),
4. The belief at stake must be key or fundamental so that its violation poses a serious risk to the health professional’s moral integrity (the “integrity criterion”).

If these criteria are to be acceptable, Neal concludes that objectors’ duties need to include respectful behavior, avoidance of placing unnecessary burdens on colleagues and provision of emergency treatment.

### Conscientious Objection to Abortion

While up to this point the discussion has focused on conscience generally and conscientious objection to the provision of treatment, in this section we now consider the literature in relation to the provision of abortion care. As indicated above, the current legal position throughout the world reflects a continuum from the free availability of abortion to its complete restriction (Center for Reproductive Rights 2013). Scotland fits into the second most liberal category.

The International Federation of Gynecology and Obstetrics (FIGO) (2006) published their own criteria for conscientious objection as:

1. To provide notice of professional services they decline to undertake on conscience grounds,
2. To refer patients for such services to other practitioners,
3. To provide such referrals timeously,
4. To provide emergency care where required.

The standards themselves, while brief, have been the subject of many commentaries and explanations, yet there is still overt polarization of the rights and responsibilities of health care providers in relation to the women’s expectations. Heino et al. (2013, p. 252) for example state that “European countries should critically assess the laws governing conscientious objection and its effects on women’s legal rights”, while conversely Pellegrino (2014) argues that a health professional’s religious values should never be placed in a secondary position to the health service’s requirements. Bowman and Schandevel (2012) concur saying that those who propose access at the expense of conscience rights could marginalize physicians who adhere to Hippocratic values. A “White Paper” which draws on the international literature from a number of disciplines attempts to sum up the issue and develop a road map for the future (Chavkin et al. 2013). The authors give clear acknowledgement to the lack of well carried out empirical research on the topic but conclude from their review that there is a growing trend towards refusal to provide certain reproductive health services, especially abortion. Acknowledging the difficulty of the situation, they recommend that a standard definition of conscientious objection should be developed together with accompanying obligations.

### Midwifery and Conscientious Objection

Much of the literature previously discussed reflects the position of the medical profession. Westeson (2013) touched on the nature of the public health services provided in many countries, but the reality is that the roles of doctors and midwives differ within those systems. This can be seen in health care systems that are privatized, as once doctors have completed all their training to become consultants, they are free to provide the services that they wish and can decline to provide those to which they have an objection without having to provide any reasons. Midwives are in a different position who, together with nurses, have been classified as “mid-level health care providers” by the WHO (2012). They are almost always employees in a field which is dominated by medicine, regardless of the funding of the health care systems in which they work. This means that the imperative for a doctor with a conscientious objection to abortion is to refer promptly to another practitioner, midwives normally do not have the flexibility to do this.

Despite this limitation, the Royal College of Midwives’ (RCM) (1997, p. 2) guidance to its members states that a midwife may have to weigh up her own position in relation to the woman’s interests and refer her care to another midwife if she sees conflicts arising due to her conscience. It does not offer suggestions as to how this can be achieved in a busy ward. Moreover, the RCM have stated that “all midwives should be prepared to care for women before, during and after a termination in a maternity unit under obstetric care” (Royal College of Midwives 1997). This statement neither considers the flexibility issue introduced above nor gives consideration to whether midwives should have the right of refusal to participate in abortion-related care.

Although, due to its statutes and to the voluntary nature of its membership, the RCM’s advice is not binding, it nonetheless carries weight in the profession. The Nursing and Midwifery Council’s (NMC) (2015) legally binding Professional Code advises nurses and midwives to think very carefully before taking the step of conscientious objection.

In the academic literature, however, the voices of midwives are generally invisible (Fleming et al. 2018). Kane (2009), however, acknowledges the change in type of treatment from surgical to medical abortions. This considers that with medical abortions, the prescription is written by a medical practitioner, but the drug is administered by a nurse, midwife or the woman herself. As the woman then experiences labor she is cared for by midwives from about the 14th gestational week. The author comments that staff members are confronted with new duties which constitutes a new challenge for ward managers. None of the professional and legal guidance offers a substantive discussion of the expectations of midwives during the labor of women who are undergoing an abortion, which is a distressing time for all concerned.

McHale (2009) suggests that the time has come to revise public policy and not to permit nurses to opt out of procedures such as abortion stating “there is a danger in allowing ‘opt-out’ to be seen as an entitlement gradually through guidance, without the legitimacy and the boundaries of such an opt-out being subject to a thorough reconsideration” (p. 1262).

She offers no suggestion as to how such a radical change could be implemented in the light of the law, but she articulates the underlying views of many which have been under-expressed in the literature.

The only research article we found that specifically concerned midwives reports a qualitative study carried out in Bern, Switzerland (Cignacco 2002). This has some limitations, in that it does not provide a detailed analysis of the ethical viewpoints of the midwives, as a commentary on this research by Newell (2002, p. 192) points out: “we do not have much of an insight into the moral reasoning of these midwives, especially in terms of their wider professional and social dialectic”. The clear lack of midwives’ voices was noted in a systematic review of reasons for exercising conscientious objection to abortion by nurse and midwives (Fleming et al. 2018). None of the final articles included in the review was written by a midwife or a nurse, implying that others may still dictate the parameters of their practice. This is also the case in Sulmasy’s (2017) article, who considered tolerance and professional judgement, and argued that “discretionary space” for a physician is needed, while ignoring that there is also a need for a “discretionary space” for other HCP, such as, for example, midwives.

### Presentation of the Case

The two midwives at the heart of the case both worked as senior midwives in the labor ward of the Southern General Hospital (SGH), Glasgow. Each of them had over 20 years’ experience in this clinical area. The dispute arose at a time during which new developments took place in both obstetrics and public health affecting many aspects of midwives’ clinical practice. As the result of a national review of duties and functions of midwives at all levels, new job descriptions for midwives throughout Scotland were issued in 2005. The job description for the senior labor ward midwives stated that “The post holder is responsible for providing clinical leadership and operational management for delivery of the midwifery service within labor wards and obstetric theatres” (Southern General Hospital 2005) (Table 1).

**Table 1:** Overview of the timeline of the case

Year	Case	
Open	*European court of human rights* Appeals court for all 47 signatory countries to the European Convention on Human Rights	
2014	*Supreme court* GGCHB Counter Appeal that ruled against the midwives	
2013	*Inner House of Court of Session* Ruled in favor of the midwives	
2012	*Outer house of court of session Edinburgh* Ruled against the midwives	
2011	Grievance process within the GGCHB was not upheld	
2009	Letter from SGH management to Mary Doogan expressing the expectation that abortion related care is provided by the midwives	
	Numerous attempts of the midwives failed to resolve the conflict informally	
	Midwives came into conflict with SGH because CO appeared to becoming increasingly difficult	
2005–2010	Restructuring processes at SGH	
	CO was possible at SGH	

Prior to signing the job descriptions, the two midwives reconfirmed with their managers their right to conscientious objection to abortion. Of particular concern were three aspects of the new job descriptions which stated: “Provide clinical leadership, guidance and support to staff and be responsible for ensuring their supervision, training and education; direct/assist with the planning and delivery of clinical teaching of student midwives and nurses, qualified midwives and other learners and consult with medical staff in identifying and solving problems in patient care which lie outwith the scope of autonomous midwifery practice” (Southern General Hospital Glascow 2005). Each of these statements made it clear that the senior midwife on duty on the labor ward was expected to keep abreast of all that is happening with all the women in the ward during their shift and ensure good clinical care.

At the time of the changes, very few abortions were carried out in the labor ward but women’s health services in the city of Glasgow were restructured in 2007. By 2010, this resulted in approximately 160 late abortions, after the 16th week of pregnancy, taking place in the labor ward, a number that in subsequent years has steadily increased. During this procedure, which might last up to 24 h or more, a woman’s labor is induced and she requires one to one care from an experienced midwife. Due to such large numbers it was not possible for two such senior midwives to organize their rosters not to be on duty during the times at which women would be undergoing abortions. Numerous attempts were made by the midwives to resolve the issue informally and then formally by letter with their line managers, and while verbal support was expressed, a written response was slow to come. Finally, in 2009, 18 months after the issue was first raised with management, a letter was written by a manager which stated that their right to conscientious objection was upheld but “you must continue to provide care for women undergoing termination of pregnancy” (Letter from labor ward manager to Mary Dougan 2009). Due to its ambiguity, this did not offer a way forward, so legal advice was sought by both sides and a formal grievance procedure commenced.

### The Midwives Formal Grievance

The midwives’ formal grievance, lodged on 8 September 2009, was not upheld, the employer’s response being that the 1967 Act did not confer on the petitioners any right to refuse to delegate, supervise and/or support staff in the provision of care to patients undergoing medical termination of pregnancy. The midwives appealed, citing direct involvement with the procedure through allocation of staff on each shift and the necessity for their supervision. This appeal was also rejected reiterating the same grounds. The midwives next appealed to the Health Board. In their petition, they explained that the matter of supervision and delegation referred to above was outstanding and required clarification. Dealing with the case were the GGCHB’s Senior Nurse and Chief Executive Officer with legal advice from the Board’s solicitor. GGCHB’s decision was issued on the 14th June 2011 by letter which advised:

It is the view of the Panel that delegating, supervising and/or supporting staff who are providing care to patients throughout the termination process does not constitute providing direct 1:1 care and having the ability to provide leadership within the department is crucial to the Roles and Responsibilities of a Band 7 midwife therefore this part of your grievance is not upheld. (Greater Glascow and Clyde Health Board 2011).

## The Scottish Courts

Following exhaustion of the grievance process within the Greater Glasgow and Clyde Health Board, the midwives petitioned the Court of Session in Edinburgh in January 2012 for a judicial review based on Section 4(1) of the Abortion Act and Article 9 of the European Convention on Human Rights (ECHR) (1950). The main question for consideration remained as in the grievance: “are the respondents [the hospital] entitled to require them to delegate, supervise and support staff in the treatment of patients undergoing termination of pregnancy?”

The midwives’ petition included 13 aspects of their work in which they were required to engage with the process of procuring abortion:

1. Ensuring appropriate management of resources,
2. Providing a detailed handover on each patient within the labor ward,
3. Allocating appropriate staff to patients,
4. Providing guidance and support to staff caring for women undergoing abortions,
5. Accompanying obstetricians on ward rounds,
6. Responding to requests for assistance including the emergency pool,
7. Acting as the first point of contact for the midwives providing direct care,
8. Ensuring the provision of relief for compulsory breaks for midwives providing the direct care,
9. Being present to support and assist if medical intervention is required, for example, instrumental delivery with forceps,
10. Monitoring the progress of patients to ensure that any deviations from normal are escalated to the appropriate staff level, e.g., an obstetrician,
11. Communicating with other professionals,
12. Providing care in emergency situations,
13. Ensuring that the family is provided with appropriate support.

The petition was heard in the Outer House by a single judge, whose ruling referred to the moral argument against abortion and that in this case the midwives’ objection was due to their membership of the Roman Catholic Church whose teachings were aimed at the preservation of life (Doogan et al. 2012). However, the judge did not make any reference to the *Code of Canon Law*, which is the universal law binding on Catholics throughout the world, where the penalty for abortion is automatic excommunication (CIC 1986). Similarly, as aforementioned, no reference was made at any time to the debates in the academic literature.

In her ruling against the midwives released on 29 February 2012, the judge gave weight to the consideration of the phrase “participate in any treatment authorised by this [Abortion] Act and concluded that the word “treatment” was being used “to denote those activities which directly bring about the termination of the pregnancy”(*Doogan, M. & Wood, C. v Greater Glasgow and Clyde Health Board* 2012). She went further, discussing the meaning of participation as “taking part in” but she restricted herself to a narrow view of participation, and as the midwives were not being required to play any direct part in bringing about the termination of pregnancy, they were not being asked to “participate in any treatment authorised by this Act” (*Doogan, M. & Wood, C. v Greater Glasgow and Clyde Health Board* 2012). She perceived their role as purely supervisory and administrative where no direct contact with the woman was required.

A supporting reason for her judgement was that the right of conscientious objection is not unqualified. In this case, she ruled that the midwives could not have thought that in accepting the job description their statutory rights would prevent them from having to provide treatment to terminate pregnancy in all circumstances. Additionally, they had accepted the role of labor ward coordinators and it was this same job content to which they were, she ruled, belatedly taking objection.

The midwives appealed against the judgement of the Outer House in the Inner House, which was heard by three judges. In the background to the appeal the same 13 scenarios submitted by the midwives were discussed and it was acknowledged that in the previous hearing GGCHB accepted each of these would have to be decided on a day to day basis as any of them could involve direct contact with women having abortions. A new argument put forward by the midwives was that care for women undergoing abortions was not something that took place at a defined time but involved the bringing together of many factors, including intrapartum and post-partum monitoring and care as well as drug administration, many of which were ultimately dependent on each woman’s physiological and psychological reactions (Doogan et al. 2013).

The midwives’ submission to the Inner House reiterated their acceptance of the need to provide care in emergency situations where the woman’s life was in jeopardy. They contended, however, that the initial judgement of the applicable paragraphs of the Abortion Act was erroneous and that a broader view had to be taken as comprehensively stated by Lord Diplock who in a landmark 1981 judgement ruled in favor of the Royal College of Nurses (RCN) stating:

If ‘termination’ or ‘terminated’ meant only the event of miscarriage and not the whole treatment undertaken with that object in mind, lack of success which apparently occurs in one or two per cent of cases, would make all who had taken part in the unsuccessful treatment guilty of an offence under section 58 or 59 of the Offences Against the Person Act 1861. This cannot have been the intention of Parliament (Royal College of Nursing versus Department of Health and Social Security (DHSS) 1981).

In the present appeal, the focus was solely on section 4(1) of the Abortion Act, Article 9 of ECHR being “not insisted in” (1967). The midwives’ evidence concluded that:

by giving an exemption to those with a conscientious objection, the state has avoided conflict between the law and religious beliefs. Conscience and belief took precedence over law by the exemption from duty and section 4(1) should be interpreted in such a way as to allow the reclaimers to be true to their beliefs while remaining respectful of the law. (*Doogan, M. & Wood, C. v Greater Glasgow and Clyde Health Board* 2012)

Conversely, GGCHB’s submission continued to argue in favor of a narrow interpretation of the words “participate” and “treatment” contending that any broader interpretation would lead to an impossible situation in the provision of care. Additionally, it would impose extra costs on the employers, a dual system of care and an increased burden on those staff with no conscientious objection to abortion.

In their ruling released on 24 April 2013 in favor of the midwives, the three judges were unanimous in all points. They concurred that the conscience clause applied to all provisions in which abortion could be legally carried out. Likewise, they highlighted that professional advice, such as that given by the RCM, is generally in the form of non-legally enforceable guidelines. Similar advice given by the Nursing and Midwifery Council (NMC 2013) the statutory regulatory body for all nurses and midwives in the UK, although binding, was also highlighted by the judges as misleading to the professions.

The judgement drew comparisons with a case which had ruled against a medical secretary who objected to having to send letters to women with appointments for abortion (*Salford Area Health Authority v Janaway*^1988^). While ruling against the petitioner in that case, the judges showed a clear difference between all clinical staff involved and others such as secretaries suggesting that the use of the words “actually participate” applied to the midwives in the present case as they could be “taking part in treatment administered in hospital or other approved place in accordance with Section 1(3), for the purpose of terminating pregnancy” (*Salford Area Health Authority v Janaway* 1988). While sympathetic to the position of midwives (and others) who did not have conscientious objection to abortion, the judges found no evidence submitted in favor of GGCHB’s arguments concerning additional costs and compromised patient safety.

In their penultimate paragraph, the judges reversed the earlier judgement stating that “the right [to conscientious objection] is given because it is recognized that the process of abortion is felt by many people to be morally repugnant” (*Salford Area Health Authority v Janaway* 1988). The judges therefore adopted a wide interpretation of “treatment” and “participation” as consistent with the reasoning which allowed such an objection in the first place and that it should extend to any involvement in the process of treatment, the object of which is to terminate a pregnancy.

## The Supreme Court

GGCHB’s counter appeal was heard by five judges at the UK Supreme Court, London^2^ on 11 November 2014. In addition to the solicitors for the midwives and GGCHB, two interveners, the RCM and the British Pregnancy Advisory Service (BPAS), an abortion agency, were invited to make representation. The judges established that, “it will immediately be apparent that the question in this case, and the only question, is the meaning of the words ‘to participate in any treatment authorised by this Act to which he has a conscientious objection’” (*Greater Glasgow Health Board (Appellant) v Doogan and Another (Respondents)* 2014).

In their judgement, the changes in abortion provision were much more clearly articulated than in any of the previous hearings. Of particular interest to the judges was the change from surgical to medical termination of pregnancy^3^ focusing on whether “treatment” in the Abortion Act was synonymous with “termination”. In a landmark case, it was conclusively ruled that “termination of pregnancy” could only mean the whole process of treatment and not one single part of it, thus midwives and other team members were considered participants.

It was noted that the midwives were both practicing Roman Catholics, which prohibits participation in abortion. However, the Supreme Court judges went further stating that, “They also believe that any involvement in the process of termination renders them accomplices to and culpable for that grave offence” (*Greater Glasgow Health Board (Appellant) v Doogan and Another (Respondents)* 2014). Although none of the three legal rulings refers directly to the *Code of Canon Law*, this is the first time that potential culpability was noted in relation to the Church’s Law. Again, however, none of the academic literature appears to have influenced the decision.

The evidence given by the RCM clearly stated that it believed conscientious objection should be restricted to the administration of the drugs to induce labor and should not include care of the woman during the subsequent labor or birth. The two midwives concerned subscribe to a different philosophy of care, i.e., that of continuity of care which they took to include the booking of women for the procedure and follow up care (Fleming 1998).

The judges returned to the meaning of the word “participate” and expressed the view that it is only applicable to the provision of hands on care and proceeded to test these against the 13 scenarios submitted initially by the midwives. In their findings, the judges ruled that only one of these points was covered fully by the conscience clause, that of being present to assist and support if medical intervention were required and some of the others might be covered in particular circumstances. The Health Board’s appeal was thus supported.

Concern has subsequently been expressed by both lawyers and ethicists that rather than being a landmark case the narrow interpretation of the conscience clause has not provided clear guidance for the future as there seemed to be no underpinning rationale given for its adoption (British Broadcasting Corporation 2014).

### Relevant Legal Cases

#### The RCN Case

All three UK courts referred to the Royal College of Nursing (RCN) case from 1981, which requested clarity on the legality of nurses taking part in mid-trimester abortions which were carried out by medical means.^4^ In its initial hearing, the RCN case stressed the technical nature of what in the judges’ views amounted to “performing the abortion”. Following appeal to the House of Lords^5^ the five judges ruled that by extension the term “medical practitioners” should be extended to include nurses and midwives.

The main cause for dissention in the RCN case was exactly the same as in the present one, i.e., that of what actually constituted carrying out the abortion. Lord Keith’s words summed up the general judgement:

‘Termination of pregnancy’ is an expression commonly used, perhaps rather more by medical people than by laymen, to describe in neutral and unemotive terms the bringing about of an abortion. So used, it is capable of covering the whole process designed to lead to that result, and in my view it does so in the present context. Other provisions of the Act make it clear that termination of pregnancy is envisaged as being a process of treatment. (*Royal College of Nursing v Department of Health and Social Security* *(DHSS)* 1981)

Thus, it was clearly established that abortion was in UK law considered to be a process rather than a single act or combination of acts. However, what was not established by the 1981 case was the extent to which the participation applied. The two midwives in the present case considered themselves to have direct involvement but the list of scenarios presented by the midwives, was tested against the conscience clause without direct reference to the situation of a busy labor ward.

#### Croatian Case

In Croatia, a midwife with many years of experience, known to have a conscientious objection to participating in abortion, was assigned to the operating room to assist at a surgical abortion. She advised her manager that she could not do this when it was not a procedure necessary for saving the life of the woman. Disciplinary action was initiated against the midwife, who was first suspended, then ultimately dismissed from her position although her status as a conscientious objector had previously been respected for many years. The midwife sought recourse to a powerful religious group, the *Vigilares*, which offered its backing. A well planned and executed series of press releases then saw the case making national and international headlines. Despite this, the midwifery association offered no support to its longstanding member. However, the *Vigilares* wrote a letter to the Minister of Health (2013) concerning the case and asking for his intervention. A telephone call from the Minister to the hospital’s Medical Director saw her reinstated although with fewer responsibilities.

#### Swedish Case

The other related case in Europe is currently ongoing. It involves a Swedish newly qualified midwife, who having been offered a position had it rescinded when her stance against abortion became known. After four unsuccessful attempts to gain a post as a midwife, she sought recourse in law, notifying the County Council of discrimination against her because of her religious beliefs. The Council and later the Discrimination Ombudsman found against her. Sweden, unlike most other European countries, does not have a law protecting workers’ conscientious objection and the reason given for rejecting the claim was that she was unable to fulfil the role of a midwife (*Grimmark v Landstinget i Jönköpings Län* 2014).

The midwife, with the backing of the international organization “Alliance Defending Freedom,” then submitted her case to the District Court of Jönköping where her lawyers contend that this is part of an emerging human rights’ problem in Sweden (Nordström 2014). The District Court judgement ruled against the midwife on the grounds that carrying out abortions was a necessary part of Swedish midwives' duties and thus she could not have suffered discrimination. She was, however, given leave to appeal and her case is currently awaiting judgement in the European Court of Human Rights.

While showing general consistency, both the above cases concern midwives providing hands on care throughout their shifts. GGCHB’s argument, conversely, is that the two midwives concerned are removed from the direct care provision and that the conscience clause therefore does not apply to them. The reality falls in between these two possibilities in that the midwives do not provide the direct care but could be called on to do so at any time during their shifts.

### Secondary Legislation

#### Professional Legislation

All midwives in the UK are required by law to register with the Nursing and Midwifery Council (NMC). According to its statutes, the NMC’s primary purpose is to protect the public. With regard to the quality of practice, standards, which are binding upon practitioners, have been implemented in a professional code (Nursing and Midwifery Council 2015) although the relevant version for the two midwives in the present case is that from 2008.

The Code of the NMC is clear about midwives’ duties of care to their patients. This was introduced in a document specifically on the topic of conscientious objection (Nursing and Midwifery Council 2013) after the RCN case. In this, the NMC spells out the need for careful thought before taking such a step and places accountability for any decisions related to conscientious objection in the hands of individual practitioners. This is in total contrast to the Religious Freedom Restoration Acts (1993) throughout the United States, which state that no government entity may substantially burden anyone’s exercising of their religion. The most recent edition of the NMC’s Code now includes conscientious objection thereby changing its status from advisory to obligatory.

The RCM, which as a professional body and trade union, might be expected to challenge government entities such as the NMC, offered its members no support, instead giving evidence supporting the Health Board in all three court hearings and drawing upon the Internaltional Confederation of Midwives (ICM) (2013) which lists abortion care as one of the seven essential competence domains of midwives.

The RCM also stated in the Supreme Court that it believed that abortion care only included administration of the abortifacient drugs and subsequent care was termed “ordinary and pastoral nursing care” (*Greater Glascow and Clyde Health Board (Appellant) v Doogan and Another (Respondants)* 2014). While this is consistent with previous position statements from the RCM it appears to be against the principles they profess with regard to midwifery care during the intrapartum process and to the judgement in the RCN case. There is no clarification of the phrase “ordinary nursing and pastoral care” and at this time when midwifery organizations including the RCM and ICM are very clearly stating their position that midwives are not nurses and that their remit is to provide continuity of midwifery care this appears to be a somewhat contradictory statement.

#### Canon Law

A further legislative framework that was relevant to the Glasgow midwives is that of the Catholic Church (*Code of Cannon Law* 1983). As the midwives’ objection was based upon their beliefs held as practicing Catholics, it is clear that Church legislation is also binding upon the two midwives. Yet, in the two cases in the Scottish courts, this was not raised and in the Supreme Court hearing, while alluded to, it was not afforded any in depth analysis.

As indicated in the introduction, the Roman Catholic Church is completely against abortion, focusing on the sanctity of life. The Catechism of the Catholic Church (CCC) (The Holy See 2000: 2270) classifies abortion as “intentional homicide” reiterating that “human life must be respected and protected absolutely from the moment of conception”.

In the present case, the judges in each of the three courts acknowledged that the midwives were Roman Catholic and thus abortion was contrary to their beliefs. However, following this acknowledgement, the point was not taken further and they were not openly supported by the Bishops’ Conference of Scotland, which is the Roman Catholic Church’s highest decision making body in Scotland. Neither did the *Code of Canon Law*, just as binding on all Roman Catholics as the NMC’s code of professional conduct is upon all midwives, receive further discussion. The tension between canon and civil law thus becomes immediately evident.

*The Code of Canon Law* canon 1398, however, neither defines abortion nor the term “procures” but merely refers to “*latae sententiae*” excommunication for one who procures an abortion. Thus, as soon as the abortion is procured, the penalty is effective. Such penalties, however, are difficult to control, especially as they should be judged by the persons themselves who commit the offence.

As acknowledged by Sanchis (2004), the *latae sententiae* penalty has been retained in the Code on only a few occasions and this is primarily to illustrate the gravity of the offence. CCC: 2272 uses the word “cooperation” in regard to abortion as constituting a “grave offence” and the next paragraph points out that the right of every innocent human to life is a “constitutive element of civil society and its legislation” (The Holy See 2000). However, such a right does not apply in civil law to unborn children, as the unborn child is not considered a person and therefore has no legal rights. Despite this, the penalty in the Catholic Church’s law remains that of that of *latae sententiae* excommunication.

As with the ambiguity in the present case surrounding the nature of the word “participate”, canon 1398 introduces two words which may also assume differing meanings, those of “completed abortion”. Here, the question of relevance is “what completes the abortion?” At the time of its promulgation with very few exceptions, the completed abortion could only be achieved by the surgical procedure of dilation of the pregnant woman’s cervix and manual removal of the baby from the uterus. However, in present times, while the administration of the abortifacient drugs will ultimately induce labor if given in sufficient quantities, it does not complete the abortion. Only the conclusion of the physiological process of labor with the birth of the baby and expulsion of the placenta will complete the process (Rankin 2017).

Such a scenario paints an opposing picture to that discussed in the preceding section where the RCM supported the hypothesis that conscientious objection should only apply to the administering of the medication. If the physiological hypothesis is accepted then unless medical intervention is required it would normally be the attending midwife who completes the abortion. Indeed, in some areas, to avoid confusion or culpability, the tasks are routinely divided up with one person administering the drugs and another supervising the birth process.

In referring to such possibilities, Green (2000, p. 1603) discusses the potential extent of those involved in procuring abortion. In this, he refers to canon 1329 §2 in which “accomplices” to a delict should also incur sentences “if without their assistance, the delict would not have been committed”. Pérez-Madrid (2004), however, makes the stronger point that the word “procure” means to perform or cooperate in the act of abortion, which must be carried out “with malicious intent”. Marazoa (2004) suggests that as canon 1398 is written in the singular and does not apply to the mother, recourse must be made to canon 1329. In one way, this reasoning is now dated as with possibilities of purchasing the abortifacient drug known as “the morning after pill” without prescription the “mother” may be the only person involved.

Marazoa discusses first the notion of “co-delinquency” suggesting that this term applies to physical persons who cooperate in a “single delinquent action”. It also, unlike the argument of the RCM, does not assume that a single offence can necessarily be broken up into different parts although it may have many perpetrators. She claims the most important issue is that of unity of purpose; in this case, the procuring of a completed abortion. This, she argues, has both objective and subjective elements. The objective element involves only the offence, not what might follow it, thus if this argument were to hold, a midwife providing post abortion counselling would not be considered a co-delinquent in the offence but one who provided the “ordinary and pastoral care” during the woman’s labor would be considered a co-delinquent. The subjective element of the argument is that all those involved must agree that their intention is to execute the same action.

Marazoa next introduces the forms of cooperation as total or partial. As discussed above, given the time between drug administration and the commencement of labor as well as the length of labor itself, it would be extremely difficult to say that anyone totally cooperated in abortion and so could be named as a “co-author”. It is more common amongst midwives that their participation would be considered as partial. How this is then evaluated is by considering whether the activity they carried out was necessary for the abortion to proceed or whether it would have proceeded anyway without their participation. Technically, once labor starts, an abortion could proceed with the woman unaccompanied by any health professionals. This is the situation, however, that the Abortion Act sought to overcome and against which organizations such as the WHO are now campaigning as it leads to many unnecessary maternal deaths.

Thus, with abortion being a process rather than a single act, it is not immediately clear as to whether even midwives providing the direct care are co-authors or merely accomplices to the offence. However, for the two midwives in the current case, Marazoa’s treatment of the “levels” at which accomplices can be involved is extremely helpful, as what has clearly been established throughout the various court hearings is that there is no fixed definition of those who participate in the abortion. It may be argued, for example, that a midwife who is instructed by the senior midwife on duty to care for a woman “in early labor” but who is undergoing an abortion is less of an accomplice than the senior midwife if she is not in possession of all the facts.

As discussed above, the midwives involved were required to supervise less experienced colleagues who may have been heavily reliant on them for guidance and assistance (Feltham 2014). Additionally, it is these senior midwives who allocate the staff to patients and are by this very act accomplices to the act even if not carrying it through themselves. This issue was addressed by Coriden (1986) as abortion started to become more common and managers of facilities offering the service raised questions concerning their own role. Responding to these questions, he concluded that while serious moral responsibility existed in relation to ancillary staff and managers, they did not fall under the jurisdiction of canon 1398. However, “co-agents in the procuring of abortion are all those who conspire by common intention and at the same time physically participate in the effective action or procedure that effects the abortion” (Coriden 1986, p. 657). As the nature of their job demands the senior midwives’ involvement, they clearly are considered in canon law to be accomplices.

## Discussion

A conclusion in the Inner House hearing was that the midwives had signed new job descriptions in 2005 and therefore should be aware of what the day-to-day workload would involve. A midwife’s job, however, is not fundamentally about the provision of abortion services although, as previously noted, the number of abortions in the labor ward of the SGH was climbing. As recorded in the Outer House judgement, most women are in labor ward to achieve a “joyful outcome”, i.e., the birth of their healthy child. This was supported in the Supreme Court where it was noted that abortions form a “tiny proportion” of all cases. Each of these statements provide an accurate appraisal of the proportion of late abortions in comparison with the remainder of a midwife’s work in a general sense but do not take into consideration particular clinical settings or workload associated with the procedure.

In the present case, the new job descriptions signed by the midwives formed part of a nationwide re-grading system for all midwives and were issued only after consultation with all midwives who had notified their intention to practice, the RCM and the National Health Service in Scotland. The two midwives involved were recognized by their employer as clinical experts in intrapartum care. As such, they retained their senior status in the same clinical area where they were already practicing and within the new personnel structures that were implemented by the hospital rather than being downgraded or allocated alternative posts in a different area under the GGCHB’s jurisdiction.

In itself, the new grading did not affect the right of conscientious objection, as this is applicable to all health professionals who may have to be involved with abortion. As their role did not substantially differ from that of their previous job description, they had not expected changes. However, they sought and received assurance from their employer that their rights were unchanged prior to signing. As clinical midwife specialists, their rights initially appeared to have been respected.

What gradually became a more urgent issue was that the increased number of women coming to the labor ward for abortions began to reveal practical difficulties. The Supreme Court judgement said that approximately 60 abortions/year were carried out in SGH but Statistics Scotland (2018) show that 146 late abortions were carried out in Glasgow in 2013 with all of these plus a few that are unreported taking place in labor ward at the SGH.

In the Inner House hearing, the petitioners’ 13 scenarios listed points at which the midwives might require to have direct involvement with the procedure. One involves providing care in emergency situations, a duty of all practitioners registered with the NMC. This was acknowledged by all parties in both the Inner House and Supreme Court although it is possible that the term “emergency situations” could be construed differently by different people, just as clause 1 also has a wide range of interpretations by different medical practitioners.

The Supreme Court judges took the view that only one of the scenarios submitted by the midwives was fully covered by the conscience clause: that of being present to assist and support if medical intervention were required. We contend, however, that five of the remaining clauses completely concern the provision of direct care during the labor, by the senior midwives, although points one and two on the list below could be seen to be synonymous. These are:

1. Ensuring appropriate management of resources,
2. Allocating appropriate staff to patients,
3. Responding to requests for assistance including the emergency pool,
4. Acting as the first point of contact for the midwives providing direct care,
5. Monitoring the progress of patients to ensure that any deviations from normal are escalated to the appropriate staff level, e.g., an obstetrician.

The first three points from the above list involve the support of junior staff. Allocation of appropriate resources, then, is done using the best available option. The use of “bank” staff employed on zero hours contracts to work as required often ensures that sufficient numbers of midwives are available but that skill mix is not always what is required by the clinical picture, which in labor wards constantly changes. Thus, the senior midwives have little option but to work with and act as a resource for people. It is often the senior midwife who has to step in and advise on or even provide the direct care as she is the only one who does not have other clinical commitments or who has the necessary expertise. Consequently, there can be no guarantees that some form of participation in abortion related care will not be required of the labor ward coordinator and employers have not provided a backup plan for such situations.

Having experienced a drop in staff/patient ratios between 2005 and 2010, there is often one, very inexperienced, midwife working between several women. Frequently women undergoing abortions may be in a position where they simultaneously require one to one care making it necessary for someone else to step in and provide this. An account of a “normal day” in a [different] labor ward in Scotland illustrates this beautifully (Page 2014, p. 31) “you’ve got three normal labourers, two inductions, a forceps in theatre, one in HDU [high dependency unit], two delivered and two electives with five midwives”. As the senior midwife allocates her staff, she tells each “just call me if you’ve got any worries”.

Likewise, mentoring of junior midwives in unfamiliar situations is expected of all senior staff in order to provide a safe level of care to all women. During their midwifery education, students are advised to gain experience of women undergoing abortions but it is dependent on their mentors and the availability of clinical experience as to how much prior experience they may have had. Thus, even if they are willing to provide the care, they may not have the competence or confidence to do so and again the senior midwife will be required to provide assistance or direct care.

Finally, point five on the above list involves monitoring progress of all patients. On an initial reading this may be seen to be achieved by requesting a report from the attending midwife. However, should some untoward complication develop and reports be required such secondary evidence may be construed as negligence. As the clinical experts on duty on their shifts, personal contact with all patients is expected as ultimately the senior midwives on duty hold the responsibility for all women in the labor ward for the duration of their shift.

The senior midwives are thus in a position in which clinical expertise as well as management competence is expected. The very fact that they hold these positions is by virtue of their midwifery qualification and they could not be replaced by someone with a management qualification only.

The question posed by the courts as to whether these midwives could be required to delegate, supervise and support staff in the treatment of patients undergoing termination of pregnancy does not appeared to have taken into account the unique position of the senior midwife in labor ward. In particular, the supervision and support elements of the job involve a great deal of patient contact.

Following the Supreme Court judgement, a case conference of lawyers and ethicists was convened to discuss the outcomes in terms not only of the present case but also as a precedent for future cases. While the participants each held their own respective positions on the rights or wrongs of abortion, the unanimous finding of the conference was that the judgement was not well reasoned and could not set a precedent for future cases. In particular, neither during the hearing itself nor in the judgement that followed was the rationale for the adoption of a narrow view of “participation” provided. Likewise, the right to freedom of religion or belief inherent in the Human Rights Act and the ECHR was dismissed as a distractor instead of being admitted into the proceedings. No reason was provided for this decision (Neal 2014).

The Royal College of Midwives (RCM) has given some somewhat surprising, although consistent, evidence. Firstly, despite the RCN ruling that abortion was a whole process, the RCM’s (1997) position paper suggested that the contrary was what it expected from midwives, i.e., that the procedure of abortion was only the administration of the abortifacient drug and thus its members could only opt out of that part of the process. Not only was this a contradiction to a previous landmark court decision but it also is against its own repeated position which states that continuity of care is the most defining element of midwifery practice and is what distinguishes it from other professions.

It is noteworthy that following release of the judgement the legislative body for midwives, the NMC, has updated its Code emphasizing that the values and principles contained within are not negotiable or discretionary. The new principle 4.4 states that nurses and midwives who have a conscientious objection are obliged to tell colleagues, managers and the person receiving care and arrange for a suitably qualified colleague to take over responsibility for that person’s care. Also clearly stated within the Code are the words “you can only make a ‘conscientious objection’ in limited circumstances” (Nursing and Midwifery Council 2015). As argued previously, it is not always possible for mid-level providers to select their own replacements. Likewise there is no guidance provided as to the extent of the “limited circumstances” that they allow, leaving midwives in a vulnerable position.

As aforementioned, it is surprising that Canon Law was not raised at any of the court hearings. This may be because it is largely considered irrelevant in the UK where Catholicism is a minority religion but shows a marked contrast to the cases judged in the European Court of Human Rights, something that will need to be considered if the case is admitted there. The importance and consequence of participating in the act of abortion for the two midwives, given their faith, should have been given greater weight. As per Neal’s criteria outlined above, the midwives’ views fulfilled these and should have been considered in the deliberations.

In responding to the Supreme Court judgement, the well-known Catholic ethicist, John Haldane, stated that he felt that the judgement itself was reasonable given the focus of the deliberations (British Broadcasting Corporation 2014). However, he also suggested that it could have been resolved more satisfactorily out of court in a way that would not require individuals to be burdened with issues against their consciences. The arguments presented throughout this paper suggest that this has not yet been done in a satisfactory way and thus the question posed at the beginning of this paper “Is GGCHB entitled to require the midwives to delegate, supervise and support staff in the treatment of patients undergoing termination of pregnancy?” cannot yet be answered.

## Concluding Statement

This paper has examined the case of *Doogan and Wood versus Greater Glasgow and Clyde Health*. The case against the midwives has been concluded in the UK but the door was left open for an appeal to the European Court of Human Rights as the issue at stake had not been resolved. Should an appeal have been lodged in this court, Canon Law probably would have been taken into consideration as it has in other cases concerning freedom of religion. However, the midwives have decided not to pursue this option. The question posed by the courts and underpinning this paper was “Is GGCHB entitled to require the midwives to delegate, supervise and support staff in the treatment of patients undergoing termination of pregnancy?” While the Supreme Court’s answer was “Yes”, this paper has shown that the legal system has failed to address many of the ethical issues impacting on the job of the two midwives at the centre of this case.

The meaning of participation in abortion still remains unclear and this is now being addressed in a study, involving Valerie Flemming and Lucie Frith, in which the key deliverable will be identification of parameters for conscientious objection to abortion. The results have the potential to influence medical, midwifery, nursing and pharmacy practice as well as assisting management in these areas. The results will also be meaningful for parliamentary decisions both in the UK and devolved UK governments but also for worldwide existing legal, practical and academic challenges related to CO. The examination of this legal case undertaken in this paper underlined the need to address the existing tension between ethics and law and that research on the identification of parameters related to CO is urgently needed.

## Footnotes

1 Databases searched were from the humanities, law, religion and medical sciences using the terms “abortion”, “conscience”, “conscientious objection”, “midwives” and combinations thereof. Additional sources were church, legal and professional documents. Articles from the last 20 years were sought and followed up through their bibliographies.

2 The highest UK court which judges cases from England, Wales, Scotland and Northern Ireland, each of which have different legal systems.

3 This has gradually taken place beginning with 16.4% of abortions 1992, to 83.7% in 1997 (ISD 2018).

4 The medically induced abortions, which then were a new development in medical science, used a different procedure from what is now common practice but nonetheless induced labor and midwives as well as nurses were required to care for the women during their labors.

5 The predecessor of the Supreme Court.

## References

[CR1] *Abortion Act*. (1967). London: Her Majesty’s Stationary Office. http://www.legislation.gov.uk/ukpga/1967/87/pdfs/ukpga_19670087_en.pdf. Accessed 19 June 2019.

[CR2] Antommaria A (2010). Conscientious objection in clinical practice: Notice, informed consent, referral, and emergency treatment. Ave Maria Law Review.

[CR3] Baker R (2009). Conscience and the unconscionable. Bioethics.

[CR4] Bowman M, Schandevel C (2012). The harmony between professional conscience rights and patients´ right of access. Phoenix Law Review.

[CR5] British Broadcasting Corporation (2014). *Scotland 2014. Wednesday 17 December 2014.*

[CR6] Caston V (2004). Aristotle on conscientiousness. Mind.

[CR7] Center for reproductive rights (2013). *The world’s abortion laws. Map 2013 update*. http://www.reproductiverights.org/sites/crr.civicactions.net/files/documents/AbortionMap_Factsheet_2013.pdf. Accessed 19 June 2019.

[CR8] Chavkin W, Leitman L, Polin K (2013). Conscientious objection and refusal to provide reproductive healthcare. International Journal of Gynecology and Obstetrics.

[CR9] Cignacco E (2002). Between professional duty and ethical confusion: Midwives and selective termination of pregnancy. Nursing Ethics.

[CR10] *Code of Canon Law* (1983/1989). Latin-English Edition. Washington DC: Canon Law Society of America.

[CR11] Coriden J (1986). Commentary on canon 1398: Canonical penalty for abortion as applicable to administrators of clinics and hospitals. The Jurist.

[CR12] Council of Europe (1950). *The European Convention on Human Rights.* http://www.hri.org/docs/ECHR50.html. Accessed 19 June 2019.

[CR13] Curlin F, Lawrence R, Chin M, Lantos J (2007). Religion, conscience, and controversial clinical practices. New England Journal of Medicine.

[CR15] *Doogan, M. & Wood, C. v Greater Glasgow and Clyde Health Board* (2012). Case P876/11. Outer House. Court of Session Edinburgh, 29 February. https://www.scotcourts.gov.uk/search-judgments/judgment?id=6fb28aa6-8980-69d2-b500-ff0000d74aa7. Accessed 19 June 2019.

[CR16] *Doogan, M. & Wood, C. v Greater Glasgow and Clyde Health Board* (2013). Case P876/11. Inner House Court of Session Edinburgh, 24 April para 13. https://www.scotcourts.gov.uk/search-judgments/judgment?id=428f8aa6-8980-69d2-b500-ff0000d74aa7. Accessed 19 June 2019.

[CR17] Feltham C (2014). The value of preceptorship for newly qualified midwives. British Journal of Midwifery.

[CR18] Fleming V (1998). Women-with-midwives-with-women: A model of interdependence. Midwifery.

[CR19] Fleming V, Frith L, Luyben A, Ramsayer B (2018). Conscientious objection to participation in abortion by midwives and nurses: A systematic review of reasons. BMC Medical Ethics.

[CR22] GGCHB letter dated 14 June 2011 to Mary Doogan and Concepta Wood.

[CR23] *Greater Glasgow and Clyde Health Board (Appellant) v Doogan and Another (Respondents) (Scotland)* (2014). Case UKSC 2013/0124. UK Supreme Court, London, 5 November. https://www.supremecourt.uk/decided-cases/docs/UKSC_2013_0124_Judgment.pdf. Accessed 19 June 2019.

[CR24] Green T, Beal J, Corriden J, Green T (2000). Commentary on Canon 1329. New commentary on the Code of Canon Law.

[CR25] *Grimmark vs Landstinget i Jönköpings Län* (2014). Case Number 19760930-2406. Alliance Defending Freedom Amicus Brief to District Court.

[CR26] Gutmacher Institute (2018). Abortions: Fact sheet. https://www.guttmacher.org/fact-sheet/induced-abortion-worldwide. Accessed 19 June 2019.

[CR27] Heino A, Gissler M, Apter D, Fiala C (2013). Conscientious objection and induced abortion in Europe. The European Journal of Contraception and Reproductive Health Care.

[CR29] Information Services Division Scotland (2018). *Abortion Statistics year ending 31 December 2017.* https://www.isdscotland.org/Health-Topics/Sexual-Health/Publications/2018-05-29/2018-05-29-Terminations-2017-Report.pdf. Accessed 19 June 2019.

[CR30] International Confederation of Midwives (2013). *Essential competencies for basic midwifery practice.* http://internationalmidwives.org/what-we-do/education-coredocuments/essential-competencies-basic-midwifery-practice/. Accessed 19 June 2019. 10.1016/j.midw.2011.03.005 21601321

[CR31] International Confederation of Midwives (2014). *Position Statement: Collaboration for healthy partnerships.* http://www.internationalmidwives.org/assets/uploads/documents/Position%20Statements%20-%20English/Reviewed%20PS%20in%202014/PS2008_002%20V2014%20Collaboration%20and%20Partnerships%20ENG.pdf. Accessed 19 June 2019.

[CR32] International Federation of Gynaecology and Obstetrics (2006). *Conscientious objection: position statement. *http://www.figo.org/sites/default/files/uploads/OurWork/2006%20Resolution%20on%20Conscientious%20Objection.pdf. Accessed 19 June 2019.

[CR33] Kane R (2009). Conscientious objection to termination and pregnancy: the competing rights of patients and nurses. Journal of Nursing Management.

[CR34] Letter dated March 5 2009 from labor ward manager to Mary Doogan.

[CR35] *Letter from Udruga Vigilare Dr. sc. Vice John Batarelo to Prof. dr. sc. Rajko Ostojić Croatian Minister of Health* translated Teja Zaksek.

[CR36] Marazoa Á, Marzoa Á, Miras J, Rodriguez-Ocafla R (2004). Commentary on Canon 1329. Exegetical commentary on the Code of Canon Law Vol IV/I.

[CR37] McHale J (2009). Conscientious objection and the nurse: a right or a privilege?. British Journal of Nursing.

[CR38] Neal M (2014). The scope of the conscience-based exemption in section 4(1) of the Abortion Act 1967: *Doogan and Wood v NHS Greater Glasgow Health Board* [2013] CSIH 36. Medical Law Review.

[CR39] Neal, M. (2015). In good conscience: Conscience-based exemptions and proper medical treatment. In *Paper presented at a seminar in the Law School University of Sheffield, January.* 10.1093/medlaw/fwv007 25944894

[CR40] Newell C (2002). Response to Cignacco. Nursing Ethics.

[CR41] Nordström, R. (2014). *Letter from Ruth Nordström to Ombudsman for discrimination, Jönköping re Ellinor Grimmark*. 21 May.

[CR42] Nursing and Midwifery Council (2013). *Conscientious objection by nurses and midwives.* http://www.nmc-uk.org/Nurses-and-midwives/Regulation-in-practice/Regulation-in-Practice-Topics/Conscientious-objection-by-nurses-and-midwives-/. Accessed 19 June 2019.

[CR43] Nursing and Midwifery Council (2015). *The Code: Professional standards of practice and behavious for nurses and midwives. *http://www.nmc.org.uk/globalassets/sitedocuments/nmc-publications/revised-new-nmc-code.pdf. Accessed 19 June 2019.

[CR44] Page M, Mander R, Fleming V (2014). Midwifery care with women in labour in an institution. Becoming a midwife.

[CR45] Paul VI Pope. (1968). Humanae Vitae. In A. Flannery (Ed.), *The Vatican collection, Vatican Council II the conciliar and post conciliar documents *(pp. 397–416). New York: Costello Publishing Company.

[CR46] Pellegrino E (2014). La conciencia del medico, cláusulas de conciencia y creencia religiosa: una perspectiva católica. Cuadernos de Bioética.

[CR47] Pérez-Madrid F, Marzoa Á, Miras J, Rodriguez-Ocafla R (2004). Commentary on canon 1398. Exegetical commentary on the Code of Canon Law Vol IV/I.

[CR48] Pius XII, Pope (1951). Nature of their profession. Allocution to Italian midwives, http://www.ewtn.com/library/PAPALDOC/P511029.HTM. Accessed 19 June 2019.

[CR51] Rankin J (2017). Physiology in Childbearing with Anatomy and related Biosciences.

[CR52] *Religious Freedom Restoration Act (1993).* Pub. L. No. 103-141, 107 Stat. 1488.

[CR53] Royal College of Midwives (1997). *Position Paper No.17, Conscientious Objection*. London: RCM. April.

[CR54] Royal College of Midwives (2014). *High quality midwifery care.* https://www.rcm.org.uk/sites/default/files/High%20Quality%20Midwifery%20Care%20Final.pdf. Accessed 19 June 2019.

[CR55] *Royal College of Nursing v Department of Health and Social Security (DHSS)* (1981). Case number HL/PO/JU/18/241. http://www.bailii.org/uk/cases/UKHL/1980/10.html. Accessed 19 June 2019.

[CR56] *Salford Area Health Authority v Janaway* (1988). Case UKHL 17. http://www.bailii.org/uk/cases/UKHL/1988/17.html. Accessed 19 June 2019.

[CR57] Sanchis J, Marzoa Á, Miras J, Rodriguez-Ocafla R (2004). Commentary on Canon 1314. Exegetical commentary on the Code of Canon Law Vol IV/I.

[CR58] Southern General Hospital (2005). Job description midwife.

[CR59] St. Thomas Aquinas (ca. 1270) *Summa Theologica first part. Question 79 article 13*. http://www.newadvent.org/summa/1079.htm#article13. Accessed 19 June 2019.

[CR60] Statistics Department Scotland (2018). Report of abortion statistics for 2013. https://isdscotland.scot.nhs.uk/Health-Topics/Sexual-Health/Publications/2014-05-27/2014-05-27-Abortions2013-Report.pdf?26597231627. Accessed 19 June 2019.

[CR61] Sulmasy D (2008). What is conscience and why is respect for it so important?. Theoretical Medicine and Bioethics.

[CR62] Sulmasy D (2017). Tolerance, professional judgment and the discretionary space of the physician. Cambridge Quarterly of Healthcare Ethics.

[CR63] The Holy See (2000). Catechism of the Catholic Church (popular and definitive edition).

[CR64] Weinstock D (2014). Conscientious refusal and health professionals: Does religion make a difference?. Bioethics.

[CR65] Westeson J (2013). Reproductive health information and abortion services: standards developed by the European Court of Human Rights. International Journal of Gynecology and Obstetrics.

[CR66] Wicclair M (2000). Conscientious objection in medicine. Bioethics.

[CR67] World Health Organization (2012). *Safe abortion: Technical and policy guidance for health systems*. Geneva: WHO. http://apps.who.int/iris/bitstream/10665/70914/1/9789241548434_eng.pdf?ua=1. Accessed 19 June 2019. 23700650

[CR68] Zampas C (2013). Legal and ethical standards for protecting women’s human rights and the practice of conscientious objection in reproductive healthcare settings. International Journal of Gynecology and Obstetrics.

